# Hormonomics profiles revealed the mechanisms of cold stratification in breaking the dormancy during seed germination and emergence process of Polygonatum sibiricum Red

**DOI:** 10.1080/15592324.2024.2447460

**Published:** 2024-12-29

**Authors:** Haiqing Liu, Jie Yuan, Hanjin Wu, Xiaobin Ou, Zhengkun Liu, Xiuli Liu, Shuyan He

**Affiliations:** a School of Agriculture and Bioengineering, Longdong University, Qingyang, Gansu Province, China; b Gansu Key Laboratory of Protection and Utilization for Biological Resources and Ecological Restoration, Qingyang, Gansu Province, China; c College of Medical Science, Longdong University, Qingyang, Gansu Province, China

**Keywords:** *Polygonatum sibiricum* Red, Hormonomics, seed dormancy, seed germination, seed emergence

## Abstract

*Polygonatum sibiricum* Red, known as Huangjing in Chinese, is a perennial plant valued in traditional Chinese medicine and is a nutritional food ingredient. With increasing market demand outpacing wild resource availability, cultivation has become essential for sustainable production. However, the cultivation of *P. sibiricum* is challenged by the double dormancy characteristics of seeds, which include embryo and physiological dormancy. This affected the germination of seeds and the establishment of seedlings. This study investigates the role of plant hormones in breaking seed dormancy and regulating germination and emergence in *P. sibiricum*. We found that cold stratification at 4°C for over 70 d significantly alleviates seed dormancy, associated with changes in endogenous hormone levels. Auxin, gibberellin, abscisic acid, cytokinin, salicylic acid, jasmonic acid, and ethylene were identified as key players in these processes. Exogenous applications of GA3 and 2-coumarate (2-hydroxycinnamic acid) significantly enhanced seed germination, while 6-BA and GA3 promoted corm growth and development. In conclusion, our research provides insights into the hormonal regulation of seed dormancy and germination in *P. sibiricum*, offering valuable strategies for improving cultivation practices. Further studies are needed to explore the specific mechanisms of hormone interactions and to develop optimized germination and seedling establishment strategies for this medicinally important plant.

## 1. Introduction

*Polygonatum sibiricum* Red, known as Huangjing in Chinese, is a member of the *Polygonatum* genus in the Asparagaceae family.^1^ As a perennial plant, it has been treasured as a valuable herb in traditional Chinese medicine and has also gained popularity as a food ingredient.^2^ Polysaccharides, steroidal saponins, flavonoids, and alkaloids have been identified as the key bioactive compounds in the rhizomes of *P. sibiricum*, contributing significantly to its therapeutic and nutritional properties.^3–7^ Consequently, the rhizome of *P. sibiricum* holds promise for the development of innovative pharmaceuticals and health products that could address a spectrum of diseases, including diabetes, asthma, and hyperlipidemia, among others.^8,9^ Furthermore, it exhibits potential benefits such as lowering blood sugar levels, safeguarding the cardiovascular system, and offering anti-aging, anti-tumor, and anti-Alzheimer’s disease properties.^10–13^ Moreover, the rhizome of *P. sibiricum* is a rich source of various nutrients, encompassing sugars, fats, proteins, starch, carotene, vitamins, and more. These components contribute to its reputation as a nourishing and therapeutic botanical.^14^ Currently, the popularity of functional foods, such as *Polygonatum* tea, *Polygonatum* preserves, and *Polygonatum* drinks, is on the rise.^15^ This widespread usage of *P. sibiricum* Red in traditional Chinese medicines and foods has led to a substantial increase in demand for Polygonati Rhizoma.

However, in recent years, the escalating market demand for *P. sibiricum* Red has outpaced the availability of wild resources.^16^ As a result, cultivated polygonati rhizoma has emerged as the primary source to fulfill market demand, playing a critical role in ensuring the sustainable production of this valuable plant in China. How to achieve high-quality and high-yield cultivation of *P. sibiricum* under controlled conditions is a key measure for the sustainable development of the *Polygonatum*. The key to this endeavor is the technology of seedling breeding, which nurtures seeds into young plants under controlled conditions for transplantation or further growth. This technology is crucial for enabling large-scale artificial production and ensuring the continuity of this valuable resource. However, the cultivation of *P. sibiricum* is challenged by double dormancy characteristics of the seeds, which include both embryo dormancy (the embryo is not fully differentiated at seed maturity) and physiological epicotyl dormancy (the existence of growth inhibiting substances). This type of dormancy is referred to as morphological and physiological dormancy, presenting a significant hurdle to successful seed germination and seedling emergence.^17,18^ The extended dormancy period, the challenge of achieving germination, and the low germination percentage in *P. sibiricum* seeds are primarily attributed to thick seed coat and the limited water absorption capacity of the endosperm. The endosperm cells in *Polygonatum* seeds are compact and feature dense cytoplasm, leading to minimal intercellular spaces. These characteristics influence the symplastic transport of substances.^19,20^ Highly active endogenous inhibitory substances have been identified in the spermatosphere, endosperm, and seed coat of *P. sibiricum* seeds, such as phytohormones. Additionally, the increase in abscisic acid (ABA) content during seed maturation is another contributing factor to the seeds’ dormancy in *Polygonatum*.^21^

In recent years, a substantial amount of research has focused on the seed germination of *P. sibiricum*, with particular emphasis the dynamics of endogenous hormones during this process.^19–21^ Studies have shown that seeds dormancy are influenced by a complex interplay of endogenous hormones and environmental factors, such as temperature, moisture, light, and nutrient availability.^22^ The dormancy of *P. sibiricum* seeds can be relieved through cold stratification treatment,^23^ and the application 200 mg/L 6-benzylaminopurine (6-BA) has been shown to promote seed emergence.^24^ Key genes that regulate seed dormancy have been identified, including the CYP707A1 gene, which is implicated in the degradation of abscisic acid (ABA) and exhibits higher expression levels in the corm and emergence stages compared to the seed stage. Additionally, the expression of a GA2ox gene, involved in the degradation of gibberellic acid (GA), and six auxin response factors (ARF) were found to be upregulated during the corm and emergence stages.^25^ Furthermore, other metabolites have been shown to play an important role in releasing dormancy. Research indicates that the content of arginine significantly impacts the release of dormancy.^26^

Despite recent advances, the precise mechanisms by which hormonal levels influence the breaking of seed dormancy during germination and emergence are not fully understood. In our study, we demonstrated that cold stratification plays a crucial role in modulating hormone levels, thereby facilitating the release from embryo dormancy and the subsequent emergence of seedlings. This finding provides a foundational understanding of the hormonal regulation involved in the dormancy release and germination of *P. sibiricum* seeds, offering valuable insights for improving germination percentage and seedling establishment under artificial cultivation conditions.

## 2. Materials and methods

### 2.1. Plant materials and treatments

The seeds of *P. sibiricum* were harvested in September from Qingyang City in Gansu Province (36.03°N, 108.39°E). We selected fruits that had begun to soften in late September, removed the fruit skin, and chose seeds that are plump, uniform in size, and free from diseases and pests for germination experiments. Initially, the seeds were rinsed with distilled water to remove any surface debris. Following this, the seeds were treated with a 3% hydrogen peroxide solution for 15 min to disinfect them. After the hydrogen peroxide treatment, the seeds were thoroughly rinsed three times with distilled water to eliminate any remaining solution from their surface. Subsequently, the seeds were placed into 60 × 30 cm nursery trays, with one seed per hole, resulting in 400 seeds per tray. Each tray represented a replicate, and the experiment was conducted with three such replicates to ensure the reliability of the results. The seeds in tray were stored in sand at a low temperature of 4°C for periods of 0, 20, 30, 40, 50, 60, and 70 d, respectively. Then, they were moved to an air-conditioned room at 25°C to germinate. The number of germinated seeds was counted every 3 d to calculate the germination percentage. The germination percentage is defined as the proportion of germinated seeds to the total number of seeds tested. Germination was considered successful when the radicle had broken through the seed coat.

### 2.2. Hormonomics analysis of Polygonatum sibiricum Red

#### 2.2.1. Cultivation and collection of plant materials

Using the same method described above for seed disinfection and preliminary treatment, the seeds were then placed in a tray and stored in sand at a low temperature of 4°C for 70 d. Subsequently, three sequential temperature stratifications were employed to induce germination and seedling emergence in *P. sibiricum* seed: (1) warm stratification at 25°C for 4 to 8 weeks to promote radicle extrusion and cormlet formation; (2) germinated seeds with corms were subjected to cold stratification at 4°C for 60 d; (3) the cold stratified seeds were transferred to 25°C to induce seedling emergence. In this study, the following *P. sibiricum* samples were collected at different stages: mature seeds soaked for 1 d at room temperature before the 25°C stratification (Seed); germinated seeds with a corm during a warm stratification (Corm); and seeds at the seedling emergence stage during a warm stratification (Eme). Samples were collected and rapidly frozen in liquid nitrogen and then stored at −80°C until subsequent experiment and analysis. The hormone contents were determined by Meiwei Metabolic Biotechnology Co., LTD, China (http: www.metware.cn). The detailed process is given in the following subsections.

#### 2.2.2. Sample preparation and extraction

The fresh plant specimens were promptly harvested and flash-frozen in liquid nitrogen. They were then ground into a fine powder at 30 hz for 1 min and kept at −80°C for future use. A 50 mg portion of the plant material was carefully weighed into a 2 mL plastic microtube, flash-frozen in liquid nitrogen, and subsequently dissolved in 1 mL of a methanol/water/formic acid mixture (ratio 15:4:1, V/V/V). To the extract, 10 μL of an internal standard mixed solution (100 ng/mL) was introduced to serve as internal standards (IS) for quantification purposes. The mixture was vigorously vortexed for 10 min and then subjected to centrifugation at 12,000 r/min for 5 min at 4°C. The supernatant was carefully transferred to clean plastic microtubes, followed by evaporation to dryness. The residue was reconstituted in 100 μL of 80% methanol (V/V) and passed through a 0.22 μm membrane filter in preparation for subsequent LC-MS/MS analysis.

#### 2.2.3. Ultra performance liquid chromatography (UPLC) conditions

The sample extracts were subjected to analysis using an UPLC-ESI-MS/MS system, which included the ExionLC™ AD UPLC system available at SCIEX and the QTRAP® 6500+ MS system, also accessible at SCIEX. The analytical parameters were as follows: LC: the column utilized was a Waters ACQUITY UPLC HSS T3 C18 (dimensions: 100 mm × 2.1 mm i.d., particle size: 1.8 µm); solvent system consisted of water with 0.04% acetic acid (solvent A) and acetonitrile with 0.04% acetic acid (solvent B); the gradient elution program commenced at 5% B (from 0 to 1 min), ramped up to 95% B (from 1 to 8 min), maintained at 95% B (from 8 to 9 min), and finally returned to 5% B (from 9.1 to 12 min); the flow rate was set at 0.35 mL/min; the column temperature was maintained at 40°C; and the injection volume was 2 μL.

#### 2.2.4. ESI-MS/MS conditions

Linear ion trap (LIT) and triple quadrupole (QQQ) scans were conducted on a triple quadrupole-linear ion trap mass spectrometer, the QTRAP® 6500+ LC-MS/MS System, which is equipped with an ESI Turbo Ion-Spray interface. The system operated in both positive and negative ion modes and was controlled by Analyst 1.6.3 software from Sciex. The parameters for the ESI source were as follows: ion source set to ESI±; source temperature maintained at 550°C; ion spray voltage (IS) was 5500 V for positive mode and −4500 V for negative mode; curtain gas (CUR) pressure was adjusted to 35 psi. Phytohormones were analyzed using scheduled multiple reaction monitoring (MRM). Data acquisition was managed with Analyst 1.6.3 software from Sciex, while Multiquant 3.0.3 software from Sciex was utilized for the quantification of all metabolites. Mass spectrometer parameters, including declustering potentials (DP) and collision energies (CE) for individual MRM transitions, were optimized further. A tailored set of MRM transitions were monitored for each time period, corresponding to the metabolites eluting within that specific timeframe.

### 2.3. Exogenous hormone treatment experiment

To investigate the impact of exogenous hormone treatment on the germination of *Polygonatum* seeds, the seeds were treated with GA3 at concentrations of 0, 10, 20, and 30 mg/L, and 2-coumarate (2-hydroxycinnamic acid) at concentrations of 0, 500, 1000, and 1500 mg/L, with all other conditions being the same as in section 2.1. The germination percentage of seeds treated was then detected.

Furthermore, to explore the impact of exogenous hormone treatment on the emergence of *Polygonatum*, the corms were sprayed with 6-BA at concentrations of 0, 100, 200, and 300 mg/L, and GA3 at concentrations of 0, 10, 20, and 30 mg/L, Concurrently, the size of the corms, as well as the length of the buds and roots, was measured every 10 d.

### 2.4. Data analysis

Data processing and one-way ANOVA were conducted using Excel 2016 and SPSS Statistics version 19.0. Statistical differences among different treatment (seed, corm, and eme) were evaluated using Duncan’s test. Significant differences were indicated by different letters (*p* < 0.05).

## 3. Results

### 3.1. Effect of cold stratification in breaking dormancy of *P.* *sibiricum* seed during seed germination

**Figure 1.**
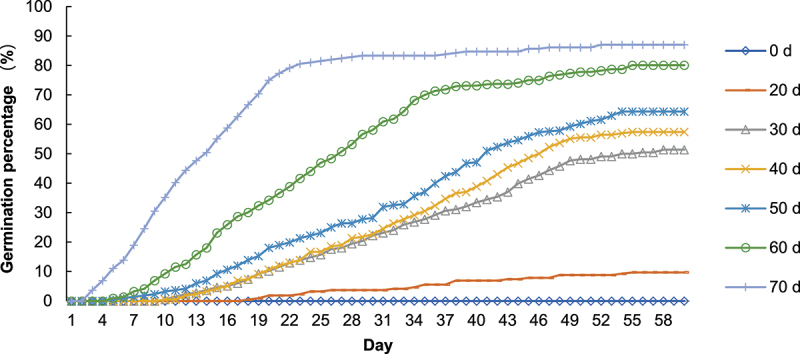
Analysis of cold stratification on breaking dormancy of *P. sibiricum* during seed germination.

To analyze the impact of cold stratification on breaking the dormancy of *P. sibiricum* seeds during germination, we conducted an experiment in which seeds were stratified in sand at a temperature of 4°C for varying durations: 0, 20, 30, 40, 50, 60, and 70 d. The results showed that cold stratification treatment had obvious effect on breaking seed dormancy (Figure 1). As the number of stratification days increased, the germination percentage also increased, with low-temperature stratification for over 30 d significantly promoting seed germination before the 25°C stratification. After 70 d of stratification treatment, the germination percentage surpassed 80% within 20 d post-sowing. However, without cold stratification, the seed germination percentage remained at zero even after 60 d. The above results indicated that cold stratification can significantly shorten the germination time.

### 3.2. Hormonomics analysis of *P.* *sibiricum* during seed dormancy, germination, and seedling emergence

**Figure 2.**
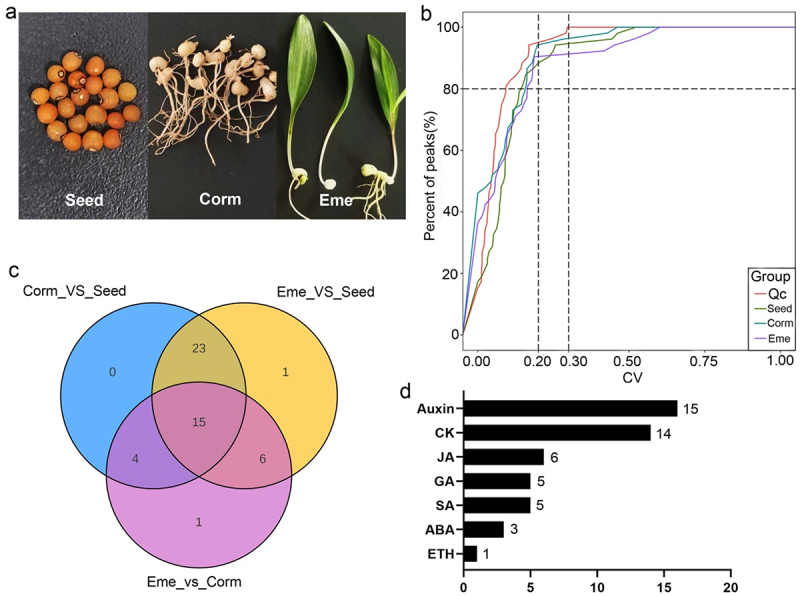
Hormonomics analysis of *P. sibiricum* during seed dormancy, germination, and seedling emergence. (a) Three main overall stages of *P. sibiricum* seeds during stratification: dormant mature seed before a warm stratification (Seed); seed germination with and cormlet formation (corm) at 25°C stratification; and seedling emergence with a leaf after transferring to 25°C (eme). (b) Coefficient of variation distribution in each group of samples. (c) Venn diagrams of results of hormone differential expression analysis. (d) Number of different expression of hormone.

**Figure 3.**
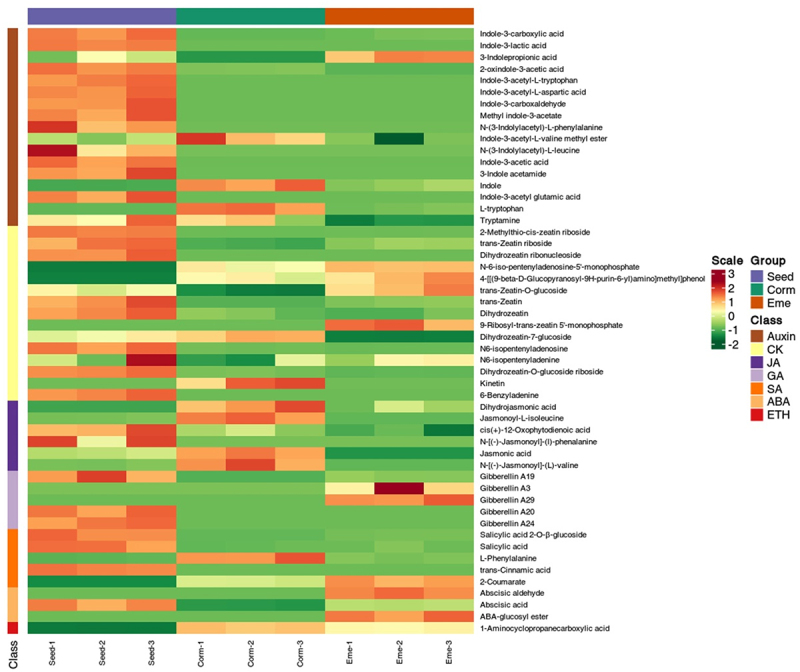
Cluster analysis heat map based on differentially expressed hormone.

Phytohormones are pivotal in governing seed germination. Consequently, we analyzed the levels of phytohormones in seeds at specific stages of germination. A contributing factor to the dormancy of *P. sibiricum* is the presence of endogenous growth inhibitors. We hypothesize that these inhibitors may be plant hormones. To test this hypothesis, we collected seeds, cormlets (which have released radicle dormancy), and seedlings (which have released epicotyl dormancy) to measure the content of endogenous hormones (Figure 2a). The results showed that the proportion of quality control samples with coefficient of variation value less than 0.2 was higher than 80%, suggesting that the experimental data were highly stable (Figure 2b). A total of 50 hormones and hormone metabolites were identified. In the corm/seed comparison group, there were 42 differential hormone expressions, and there were 26 hormones differential expressions in eme/corm comparison group (Figure 2c). Interestingly, we noted that auxin, cytokinin, jasmonic acid, gibberellin, salicylic acid, abscisic acid, and ethylene were all found to be differentially expressed according to cluster analysis (Figures 2d, 3).

**Figure 4.**
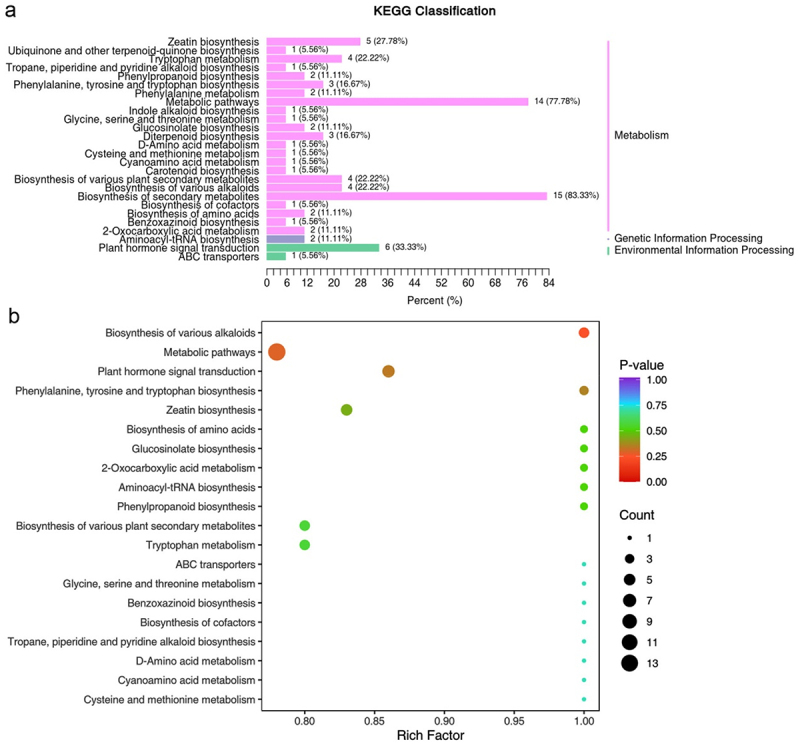
Identification of enrichment hormone. (a) Kyoto Encyclopedia of Genes and Genomes (KEGG) pathway annotation. Differential expressed hormones played important roles in metabolism and genetic information processing, according to KEGG enrichments. (b) Enrichment and distribution of differentially expressed hormone in GO functional classification bubble map.

Hormone annotation enrichment analysis revealed significant regulation of hormone in a number of metabolic processes, including the biosynthesis of secondary metabolites (83.33%), metabolic pathways (77.78%), zeatin biosynthesis (27.78%), tryptophan metabolism (22.22%), biosynthesis of various plant secondary metabolites (22.22%), and plant hormone signal transduction (33.33%), according KEGG classification (Figure 4a). Differential expression of hormones was also found to be involved in various metabolic pathways, including plant hormone signal transduction, zeatin biosynthesis, the biosynthesis of various plant secondary metabolites, tryptophan metabolism, and the biosynthesis of various alkaloids, according to Gene Ontology (GO) classifications (Figure 4b).

### 3.3. Aux/IAA is an extremely important inhibitory component during seed germination and emergence process

**Figure 5.**
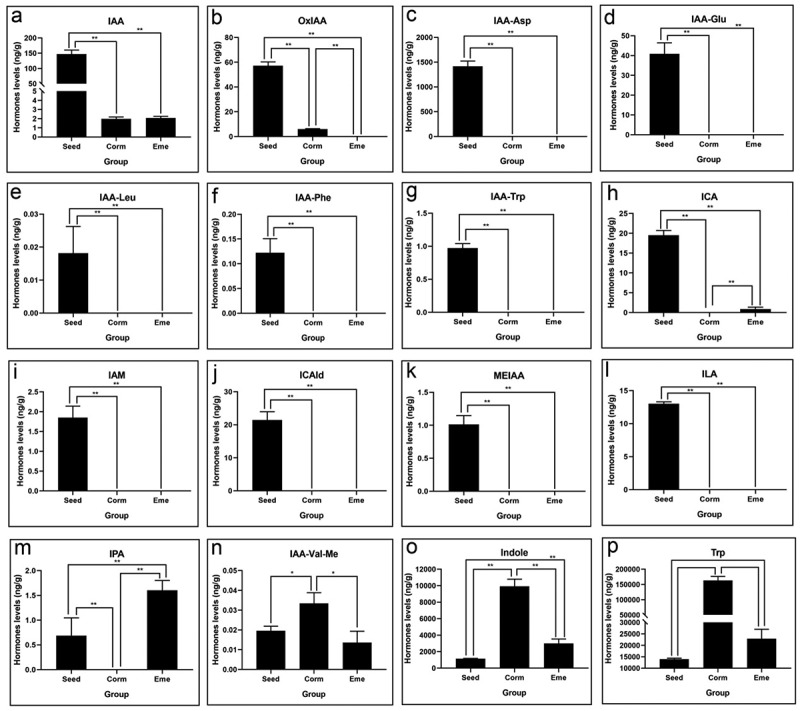
Auxin levels of of *P. sibiricum* in seed, corm, and eme during seed dormancy, germination, and seedling emergence (a-p). “*” and “**” indicate significance different at *p* < 0.05 and *p* < 0.01 level according to multiple comparison of Duncan method, respectively. The same below.

The auxin content of *P. sibiricum* during seed germination and the emergence process was analyzed. The results showed that IAA (indole-3-acetic acid) content was highest (150 ng/g) in the seed and then continued to decrease until seed germination, reaching a minimum value of 2 ng/g, but there is no significant difference in IAA content between Corm and Eme (Figure 5a). The OxIAA (oxidized IAA) content also continued to decrease until germination and emergence (Figure 5b). The conjugated auxin, which contains IAA-Asp, IAA-Glu, IAA-Leu, IAA-Phe, and IAA-Trp, followed a similar trend to IAA and OxIAA. Notably, the active forms of auxin, IAA-Asp, IAA, and OxIAA were reduced by 1417, 74, and 9.4 times in Corm than seed, respectively (Table 1). Other forms of auxin, including IAM, ICAld, MEIAA, and ILA, showed a pattern similar to IAA-Asp, with the highest content in seeds and a near-total reduction to zero in the corm and seedling (Figure 5c–l). A different trend was observed for IAA-Val-Me and the precursor substance of IAA, such as indole and Trp (tryptophan), which rapidly increased from seed to Corm and then decreased in Eme in the process of seed germination and emergence (Figure 5n–p). Conversely, ICA and IPA showed an opposite trend to tryptophan, etc (Figure 5h, m). These results show that auxin may play a negative role in seed germination and emergence in *P. sibiricum*.

**Table 1:** Functions and fold changes of selected seed dormancy/germination-related differentially expressed hormone in seed, corm, and eme during seed dormancy, germination, and seedling emergence.

Index	Compounds	Class	Corm vs. Seed	Eme vs. Corm	Eme vs. Seed
ABA-ald	Abscisic aldehyde	ABA	0.0	5.8	5.8
ABA	Abscisic acid	ABA	−15.8	6.7	−2.4
ABA-GE	ABA-glucosyl ester	ABA	0.0	6.3	6.3
ICA	Indole-3-carboxylic acid	Auxin	−19.5	0.9	−19.5
ILA	Indole-3-lactic acid	Auxin	−13.0	0.0	−13.0
IPA	3-Indolepropionic acid	Auxin	−0.7	1.6	2.3
OxIAA	2-oxindole-3-acetic acid	Auxin	−9.4	−6.1	−57.3
IAA-Trp	Indole-3-acetyl-L-tryptophan	Auxin	−1.0	0.0	−1.0
IAA-Asp	Indole-3-acetyl-L-aspartic acid	Auxin	−1417.0	0.0	−1417.1
ICAld	Indole-3-carboxaldehyde	Auxin	−21.5	0.0	−21.4
MEIAA	Methyl indole-3-acetate	Auxin	−1.0	0.0	−1.0
IAA-Phe	*N*-(3-Indolylacetyl)-L-phenylalanine	Auxin	−0.1	0.0	−0.1
IAA-Val-Me	Indole-3-acetyl-L-valine methyl ester	Auxin	1.7	−2.5	−1.4
IAA-Leu	*N*-(3-Indolylacetyl)-L-leucine	Auxin	0.0	0.0	0.0
IAA	Indole-3-acetic acid	Auxin	−74.0	1.0	−71.3
IAM	3-Indole acetamide	Auxin	−1.9	0.0	−1.8
Indole	Indole	Auxin	8.7	−3.3	2.6
IAA-Glu	Indole-3-acetyl glutamic acid	Auxin	−40.9	0.0	−40.9
TRP	L-tryptophan	Auxin	11.7	−7.1	1.6
2MeScZR	2-Methylthio-cis-zeatin riboside	CK	−383.8	3.6	−105.8
tZR	trans-Zeatin riboside	CK	−23.3	5.7	4.1
DHZR	Dihydrozeatin ribonucleoside	CK	−21.3	0.0	−21.3
iPRMP	*N*-6-iso-pentenyladenosine-5’-monophosphate	CK	1.9	1.3	2.5
pT9G	4-[[(9-beta-D-Glucopyranosyl-9H-purin-6-yl)amino]methyl]phenol	CK	0.2	1.4	0.2
tZOG	trans-Zeatin-O-glucoside	CK	−2.9	3.8	1.3
tZ	trans-Zeatin	CK	−1.8	0.3	7.3
DZ	Dihydrozeatin	CK	−3.0	−1.2	−3.6
tZRMP	9-Ribosyl-trans-zeatin 5’-monophosphate	CK	0.0	3.2	3.2
DHZ7G	Dihydrozeatin-7-glucoside	CK	1.3	−10.1	−7.6
IPR	N6-isopentenyladenosine	CK	−21.8	−1.8	−38.9
DHZROG	Dihydrozeatin-O-glucoside riboside	CK	−10.5	−3.0	−31.8
KT	Kinetin	CK	0.0	−0.1	0.0
BAP	6-Benzyladenine	CK	−78.3	0.0	−78.3
ACC	1-Aminocyclopropanecarboxylic acid	ETH	48.2	−1.3	38.4
GA19	Gibberellin A19	GA	−1.2	0.2	−1.2
GA3	Gibberellin A3	GA	0.0	1.5	1.5
GA29	Gibberellin A29	GA	0.0	8.9	8.9
GA20	Gibberellin A20	GA	−2.9	0.0	−2.9
GA24	Gibberellin A24	GA	−4.4	0.0	−4.3
H2JA	Dihydrojasmonic acid	JA	0.4	−3.6	0.1
JA-ILE	Jasmonoyl-L-isoleucine	JA	12.7	−117.8	−9.3
OPDA	cis(+)-12-Oxophytodienoic acid	JA	−2.0	−2.0	−4.0
JA-Phe	*N*-[(-)-Jasmonoyl]-(l)-phenalanine	JA	0.0	0.0	0.0
JA	Jasmonic acid	JA	2.3	−32.1	−13.7
JA-Val	*N*-[(-)-Jasmonoyl]-(L)-valine	JA	13.1	−0.5	0.0
SAG	Salicylic acid 2-O-β-glucoside	SA	−496.1	26.6	−18.7
SA	Salicylic acid	SA	−7.0	1.2	−5.6
Phe	L-Phenylalanine	SA	9.2	−5.2	1.8
t-CA	trans-Cinnamic acid	SA	−1355.8	0.0	−1355.8
2-Coumarate	2-Coumarate	SA	125.8	1.9	238.2

### 3.4. GA and ABA levels are closely related to seed dormancy and emergence in *P.* *sibiricum*

**Figure 6.**
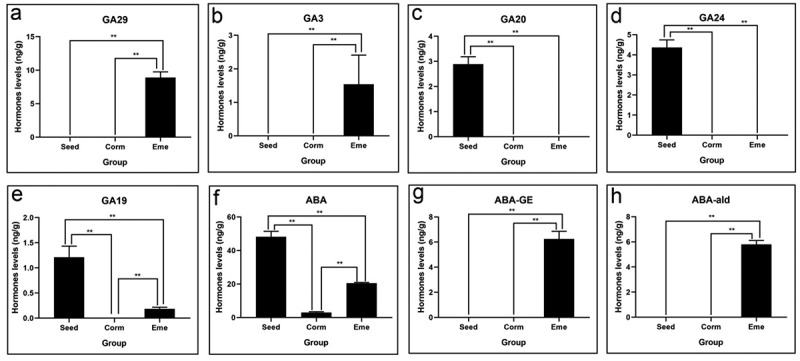
Gibberellin and abscisic acid levels of of *P. sibiricum* in seed, corm, and eme during seed dormancy, germination, and seedling emergence (a-h).

In this study, the concentrations of GA29 and GA3, important active gibberellins, steadily increased from seed to seedling. Conversely, GA20 and GA24 showed an opposite trend, while GA19 decreased from seed to corm and then increased during the seedling stage (Figure 6a-e). The levels of ABA exhibited a significant decline during the germination process and then slightly increased in the seedling stage (Figure 6f). The contents of ABA-ald and ABA-GE showed a continuous upward trend in the emergence process (Figure 6g,h). These results suggest that the levels of GA and ABA are closely related to seed dormancy and emergence in *P. sibiricum*.

### 3.5. CTK played a major role in regulating seed germination and accelerating emergence

**Figure 7.**
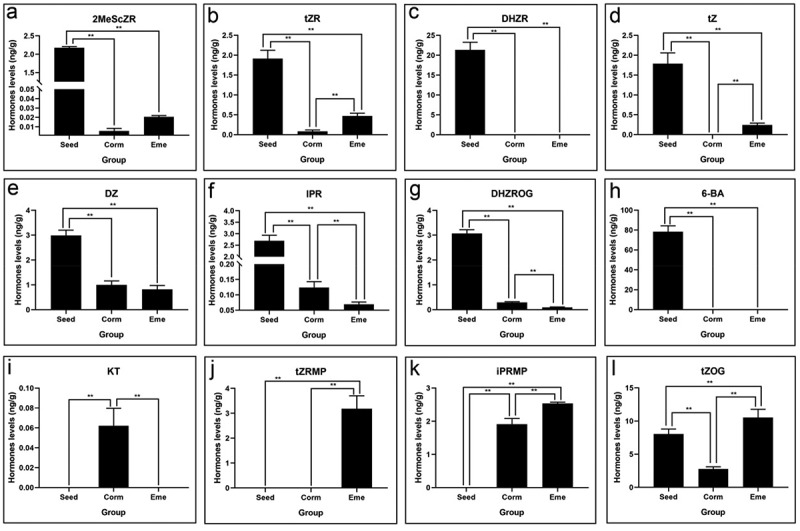
Cytokinin levels of of *P. sibiricum* in seed, corm, and eme during seed dormancy, germination, and seedling emergence (a-l).

CTK has been reported to regulate seed dormancy through interactions with ABA/GA levels. Our results indicated that 2MeSsZR, tZR, DHZR, tZ, DZ, IPR, DHZROG, 6-BA, and tZOG were found and showed a decreasing trend, primarily during the seed germination stage (Figure 7). Notably, 2MeSsZR, IPR, and 6-BA were reduced by 383.8, 21.8 and 78.3 times in corm than in seed, respectively (Table 1). In contrast, the contents of 2MeSsZR, tZR, tZ, tZRMP, iPRMP, and tZOG were found and showed an obviously increasing trend (3.6, 5.7, 0.3, 3.2, 1.3, and 3.8 times) during the emergence stage. These results suggest that CTK may play dual roles in seed dormancy and emergence in *P. sibiricum*.

### 3.6. Salicylic acid (SA) and ethylene (ETH) also participate in the seed germination and emergence process in *P.* *sibiricum*

**Figure 8.**
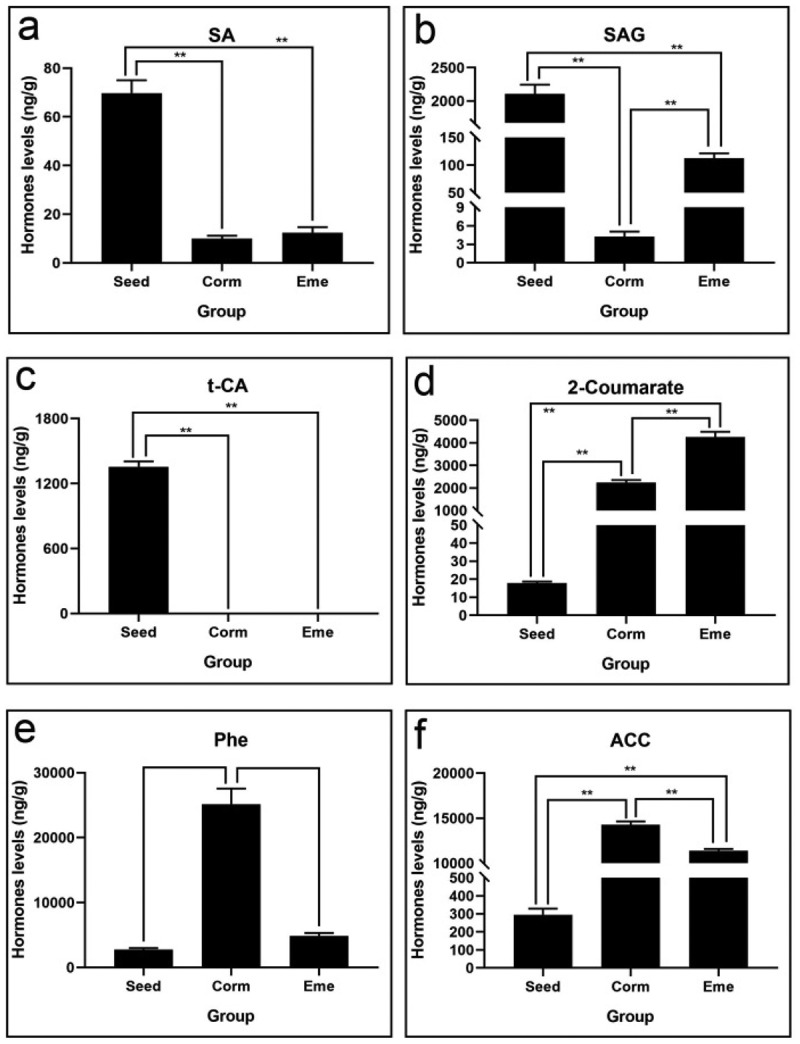
Salicylic acid and ACC levels of of *P. sibiricum* in seed, corm, and eme during seed dormancy, germination, and seedling emergence (a-f).

The results showed that salicylic acid also participates in the seed germination in *P. sibiricum*, with compounds such as SA, SAG, and t-CA sharply reduced during seed germination. They reduced 7, 496.1, and 1355.8 times in the corm than in the seed, respectively (Table 1), and SAG and 2-coumarate (2-hydroxycinnamic acid) had a marked increase in the emergence process, while Phe and ACC exhibited the exact opposite trend with SAG (Figure 8). These results suggest that SA and ETH also participate in the seed germination and emergence processes in *P. sibiricum*.

### 3.7. Jasmonic acid (JA) plays a positive role in release seed dormancy of *P.* *sibiricum*

**Figure 9.**
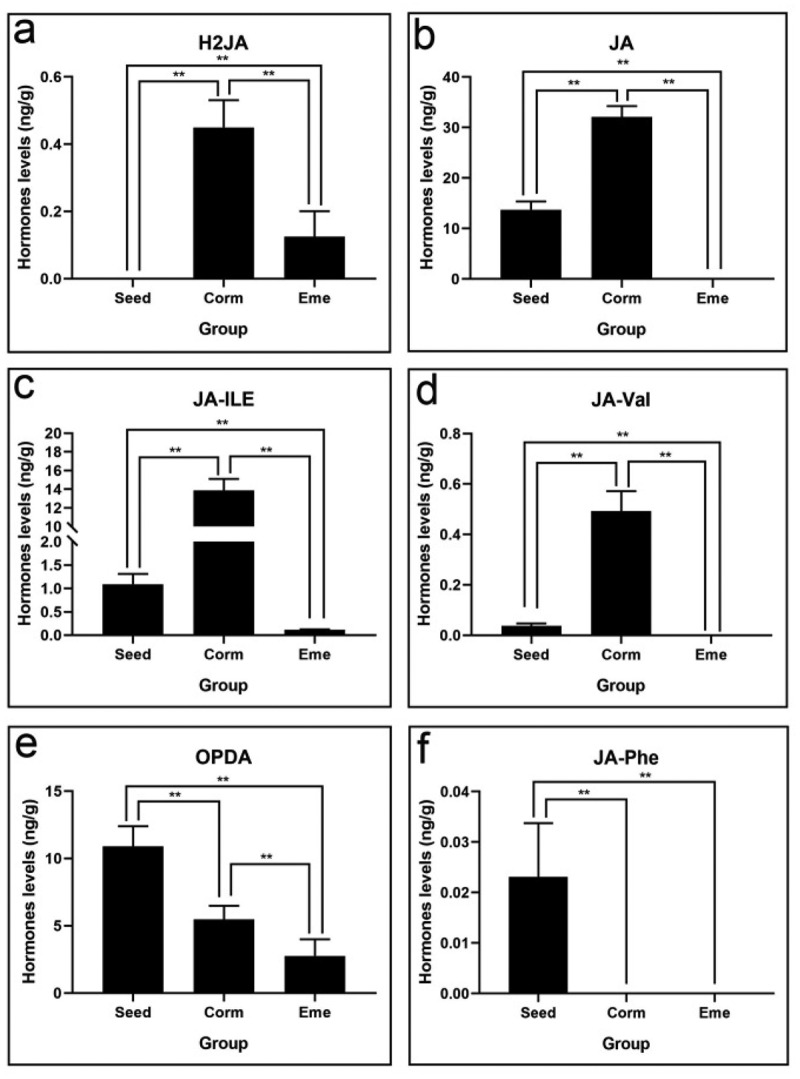
Jasmonic acid levels of *P. sibiricum* in seed, corm, and eme during seed dormancy, germination, and seedling emergence (a-f).

We also found that H2JA, JA, JA-ILE, and JA-Val have a high level in seed germination (Figure 9). Notably, JA-ILE and JA-Val increased by 12.7 and 13.7 times in corm vs. seed group, respectively (Table 1), and all the jasmonic acid including H_2_JA, JA, JA-ILE, JA-Val, and OPDA have a sharp downward trend from corm to seedling; in them, JA-ILE and JA glided 117.8 and 32 times. The results reveal that JA plays a positive role in release seed dormancy of *P. sibiricum*.

### 3.8. Effects of exogenous hormone treatment on seed germination percentage and corm growth of *P.* *sibiricum*

**Figure 10.**
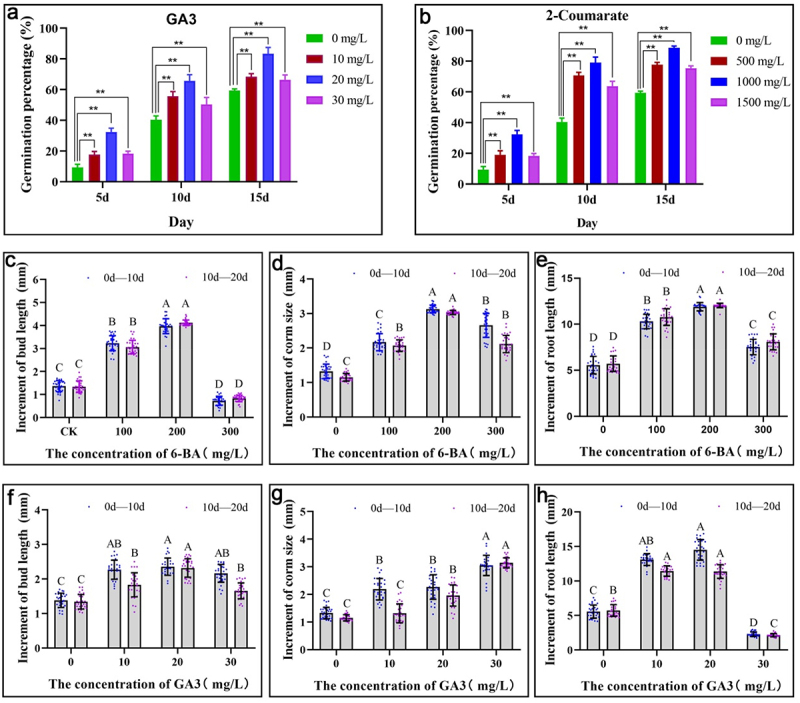
Effects of exogenous hormone treatment on seed germination percentage and corm growth of *P. sibiricum*. (a) Effects of different concentrations of GA3 on the germination percentage of *P. sibiricum* seeds. (b) Effects of different concentrations of 2-coumarate on the germination percentage of *P. sibiricum* seeds.(c-e) Increment of bud length, corm size, and root length of corm with the different concentrations of 6-BA, respectively. (f-h) Increment of bud length, corm size, and root length of corm with the different concentrations of GA3, respectively. The capital letter on the bar indicated significance different at *p* < 0.01 level according to multiple comparison of the Duncan method.

The determination of endogenous hormone levels revealed that GA3 and 2-coumarate play a significant role in promoting seed germination. To validate these findings, we subjected *Polygonatum* seeds to exogenous hormone treatments and assessed the effects of these treatments on seed germination percentage. The findings demonstrated that optimal concentrations of GA3 and 2-coumarate can significantly enhance the germination of *Polygonatum* seeds, with particularly pronounced effects observed at 20 mg/L for GA3 and 1000 mg/L for 2-coumarate (Fig 10). Additionally, the corms were sprayed with varying concentrations of 6-BA and GA3 to investigate the effects of exogenous hormone treatment on corm growth. The results indicated that appropriate concentrations of 6-BA and GA3 treatment significantly increased the growth of the corms, while also promoting the growth of buds and roots. In particular, the treatment with 200 mg/L of 6-BA and 20 mg/L of GA3 showed significant effects.

## 4. Discussion

### 4.1. Role of hormones in breaking seed dormancy

Our results indicate that cold stratification at 4°C for over 70 d significantly alleviates seed dormancy in *P. sibiricum*, suggesting that low temperatures play a crucial role in the hormonal regulation of seed dormancy. This is in line with previous studies that have shown the effectiveness of cold stratification in breaking dormancy in various plant species.^27^ The reduction in dormancy is associated with changes in endogenous hormone levels, which are key in transitioning seeds from a dormant to a germinative state.^28–30^ The hormonal profiles of *P. sibiricum* seeds during germination and emergence highlight the complex interplay of various plant hormones. Our findings show that auxin, cytokinin, jasmonic acid, gibberellin, salicylic acid, abscisic acid, and ethylene are all implicated in these processes. This aligns with the broader understanding of plant hormone function in seed physiology, where these hormones are known to regulate various aspects of seed development and germination.^31^

### 4.2. Hormonal regulation of germination and emergence

Our study identifies auxin, specifically Aux/IAA, as a critical inhibitory component during the germination and emergence of *P. sibiricum* seeds. Auxin/IAA levels are closely related to seed dormancy status, among which IAA-Asp, IAA, IAA-Glu, ICAld, ICA, ILA, and OxIAA decreased 1417-, 74-, 47.9-, 21.5-, 19.5-, 13-, and 9.4-fold changes in the seed germination (Table 1). The high levels of auxin in dormant seeds and its reduction during germination suggest a role in maintaining dormancy. This is consistent with the known inhibitory effects of auxin on seed germination in other species.^32^ The reason is that Aux/IAA is an extremely important inhibitory component that forms heterodimers with the transcription factor ARF (auxin response factor), which is responsible for regulating gene expression in the auxin signaling pathway and inhibiting the transcriptional regulatory activity of ARF.^33^ The decrease in auxin levels upon germination indicates a shift in hormonal balance, allowing for seedling emergence.

The levels of gibberellins (GA) and abscisic acid (ABA) are closely related to seed dormancy and emergence in *P. sibiricum*. Our results show an increase in GA levels from seed to seedling, which is consistent with the role of GA in promoting germination.^34^ In this study, the main active form of gibberellin, GA29 and GA3 concentration, steadily increased from seed to corm and seedling. This can be elucidated by the results that GA3oxs, and GA20oxs, showed a highly similar trend to the ascending changes in GA3 levels synchronously in *P. cyrtonema* Hua seed germination.^24^ These results provide evidence that GA may play a positive role in the release of seed germination in *P. sibiricum*. The findings of the study revealed that a dosage of 20 mg/L of gibberellic acid (GA3) significantly enhanced seed germination. Moreover, the optimal concentration of GA3 treatment was found to substantially foster bulb growth. Conversely, ABA, known for its dormancy-promoting effects,^35^ showed a significant decline during germination. This suggests a transition from a dormancy-promoting to a germination-promoting hormonal environment. In this study, ABA levels significantly decreased during germination, with a 15.8-fold reduction in corm vs. seed group (Table 1), mirroring the patterns observed in *Ginkgo biloba*,^36^ *Ginkgo biloba*,^37^ and pear.^38^ The results clearly indicated that ABA enhanced seed dormancy in *P. sibiricum*.

Cytokinins (CTK) and jasmonic acid (JA) also play significant roles in the germination and emergence of *P. sibiricum* seeds. Cytokinins, known to stimulate cell division and differentiation,^39^ showed a decreasing trend during germination and an increasing trend during emergence, indicating their dual role in these processes. CTK has also been reported to regulate seed dormancy through interactions with involved ABA/GA levels and signals.^40^ We found that 2MeScZRm, BAP, tZR, and DHZR decreased 383.8-, 78.3-, 23.3-, and 21.3-fold in corm vs. seed group, respectively. The exogenous application of 200 mg/L 6-BA promoted corm growth and development. Studies have shown that 6-BA treatment inhibited the synthesis of flavonoids. Most studies have shown that flavonoids are auxin transport inhibitors, thereby relieving the inhibitory effect on epicotyl bud differentiation of *P. cyrtonema* seeds, and the seeds could complete bud differentiation.^24^

The results also showed that salicylic acid, such as t-CA, SAG, and SA, decreased by 1355.8-, 496.1-, and 7-fold in corm vs. seed group, respectively. These results reveal that salicylic acid was positively regulated in the dormancy process of *P. sibiricum* seeds. JA plays a positive role in release seed dormancy in *P. sibiricum*. Future research will focus on the functional role of these key hormones and their interactions with each other in the dormancy release of *P. sibiricum* seeds. The exogenous application of 2-coumarate significantly enhanced seed germination, validating the role of SA hormones in promoting germination. Similarly, the application of 6-BA and GA3 promoted corm growth and development, indicating the practical application of these findings in enhancing the cultivation of *P. sibiricum*.

## 5. Conclusions

In conclusion, our study unravels the complex hormonal dynamics during the germination and emergence of *P. sibiricum* seeds. The findings underscore the pivotal roles of auxin, gibberellins, abscisic acid, cytokinins, salicylic acid, jasmonic acid, and ethylene in modulating seed dormancy (embryo dormancy and physiological dormancy) and germination. Our results provide actionable insights for enhancing the cultivation of this medicinally valuable plant, warranting further research into optimizing germination and seedling establishment strategies. Further research is warranted to explore the specific mechanisms by which these hormones interact and to develop strategies for optimizing germination and seedling establishment in *P. sibiricum*.

## Supplementary Material

Supplemental Material

## Abbreviations

The abbreviations for this article can be found at Table 1.
